# Factors influencing adherence to paediatric antiretroviral therapy in Portharcourt, South- South Nigeria

**DOI:** 10.11604/pamj.2013.16.30.1877

**Published:** 2013-09-29

**Authors:** Rosemary Ugwu, Augusta Eneh

**Affiliations:** 1 Department of Paediatrics and Child Health, University of Port Harcourt Teaching Hospital, Port Harcourt, Nigeria

**Keywords:** HIV, adherence, antiretroviral therapy, children, self report, Port Harcourt, Nigeria

## Abstract

**Introduction:**

The efficiency of antiretroviral therapy (ART) depends on a near-perfect level of patient's adherence. Adherence in children poses peculiar challenges. The aim of the study was to determine the adherence level and factors influencing adherence among HIV-infected children and adolescents in University of Port Harcourt Teaching Hospital, Nigeria.

**Methods:**

A cross-sectional survey of HIV-infected children and adolescents on ART using self-report by the caregiver/child in the past one month.

**Results:**

A total of 213 caregivers and their children were interviewed. A hundred and sixty-two (76.1%) had adherence rates ≥95%. Only 126 (59.2%) were completely (100%) adherent. The commonest caregiver-related factors for missing doses were forgetfulness 48(55.2%), travelled 22(25.3%) and drugs finished 16(18.4%), while the child-related factors were refused drugs 10(11.5%), slept 8(9.2%), and vomited 8(9.2%). Sixty-eight (31.9%) caregivers reported missing clinic visit and reasons given were travelled 18(26.5%), caregiver ill 12(17.6%) and family problems 9(13.2%). Predictors of poor adherence include mother as the primary caregiver (OR 3.32; 95%CI, 1.33-8.67), younger than 5years (OR 2.62; 95%CI, 1.30-5.31) and presence of a co-morbidity (OR 3.97; 95%CI, 1.92-8.33). Having a medication reminder strategy (OR 6.34; 95%CI, 3.04-13.31), regular clinic visits (OR 8.55; 95%CI 4.01-18.45) and status disclosure (p = 0.008) predicted a better adherence. The caregiver's age (p= 0.11), education (p = 0.86), socioeconomic status (p = 0.89), gender of the child (p = 0.84), type of ART (p = 0.2) and duration of ART (1.0) did not significantly affect adherence.

**Conclusion:**

Adherence is still suboptimal. Since barriers to Paediatric ART adherence are largely caregiver-dependent, identifying and addressing these barriers in each caregiver-child pair will improve adherence and patient outcome.

## Introduction

The Human Immunodeficiency Virus (HIV) infection remains a major public health crisis in Nigeria and has continued to spread at an alarming rate among children. In response to this pandemic, many countries including Nigeria have developed care and treatment programs. The ultimate goal of antiretroviral therapy is to achieve maximal and durable suppression of virus replication. This will in turn reduce the destruction of CD4 cells, reduce immune suppression and slow disease progression [1]. These benefits however can only be achieved through consistent adherence to antiretroviral drugs in order to maintain adequate drug levels in the body.

For HIV infection, reliable viral suppression requires a near-perfect level of adherence [2]. Unfortunately, maintaining adequate levels of adherence to antiretroviral medications has proved challenging not only for persons (especially children) living with HIV, but also for healthcare providers [1, 3] because a failing regime as a result of poor adherence will lead to increased opportunistic infections, increased hospitalization and outpatient visits and thus increased work load. Suboptimal adherence with resultant treatment failure is still common [1, 4]. Inadequate adherence to treatment is associated with detectable viral loads, declining CD4 counts, disease progression, episodes of opportunistic infections and poorer health outcomes [3, 5–8]. Non-adherence may eventually mar the dramatic improvements in HIV-related health parameters.

Antiretroviral adherence in young children and adolescents poses unique and formidable challenges. Many of them are still largely dependent on a caregiver to take their medications. Young children and adolescents may refuse to take medication especially as the reason for such medication may not have been disclosed to them. Understanding the factors that influence adherence is therefore very crucial in order for the health care provider to develop measures to support and sustain patient's adherence in the clinical care of HIV infected children. More so, there are limited paediatric antiretroviral (ARV) formulations, hence the need to ensure high levels of adherence and prevent drug resistance. Most studies in Nigeria have mainly addressed adherence in the adult population [9–11]. The aim of this survey was to determine the adherence rates and the factors influencing adherence to ARV treatment in our paediatric population.

## Methods

This was a cross-sectional survey of children and adolescents aged 3months -18years with HIV infection and who are receiving antiretroviral (ARV) drugs at the Paediatric Infectious Disease Unit of the University of Port Harcourt Teaching Hospital (UPTH). The unit provides care and treatment for all HIV exposed and infected children presenting in UPTH. Available fixed dose ARV drug combinations in our centre include zidovudine, lamivudine and nevirapine / stavudine, lamivudine and nevirapine/ zidovudine, lamivudine and Efavirenz/ Tenofovir and Emtricitabine/ and Lopinavir/Ritonavir. All the ARV drugs are given free to the patients. Adherence counseling was done for each client before ARV drugs were started and further reinforced on every clinic visit.

Readiness to receive ARV treatment was based on a number of factors including having a dedicated caregiver that will assume responsibility of administering the drugs regularly, if the mother on ARV treatment is well motivated and have a care plan based on their daily routine, demonstration of being adherent to other drugs for opportunistitic infections like antituberculous drugs or cotrimoxazole, and if there has been regular scheduled clinic visits and the caregiver demonstrates understanding of consequences of poor adherence including disease progression.

Barriers to adherence were assessed and addressed on every clinic visit for every client on ARV drug. By December 2011, about 280 children were receiving treatment with ARV drugs. HIV-exposed infants who are in care only as well as HIV-infected children who have not yet been initiated on therapy and those that have been on therapy for less than a month were excluded. Before the study, several focus group discussions and interviews were held with the caregivers during clinic attendance and support group meetings. During the focus group discussions, each member narrates their experiences and challenges with living with HIV, adhering to their drugs and the difficulties they face caring for their infected children. Solutions are proffered by other members based on their personal experiences. Each member is also expected to give a feedback on the type of care they are receiving, areas they want improvement and how this improvement would be achieved. From the outcome of these discussions, possible factors that may affect adherence were generated and used in a pilot study after which the final questionnaire for this study was designed. Information obtained from the pre-tested interviewer-administered structured questionnaire included the characteristics of the patient (age, education and occupation of the caregiver, age and sex of the child), duration of the antiretroviral therapy and their understanding of the implication of poor adherence. The type of ARV drug combination regimens prescribed, their adherence level to ARV drugs, the factors of poor adherence, other co-morbidities as well as disclosure issues were also obtained. The social class was determined by using the occupation and educational status of the caregiver [12]. Clients who did not attend clinic within the study period were contacted on phone and responses from the caregivers were entered into the questionnaire.

Adherence rates were measured by means of self-reporting by the caregivers/patients It was assessed as the number of doses missed during the last 3 days (three-day recall), the last 7 days (7 days recall) and the last one month (one month recall). Adherence score was expressed as the proportion (%) of tablets taken compared to prescribed tablets. An adherence rate of ≥95% was accepted as optimal adherence whereas patients were considered non-adherent if the total dose of antiretroviral drug taken was less than 95% of that prescribed. Patients identified as “non-adherent” were seen weekly for three weeks and counseled on drug adherence. They were reassessed on the fourth week using a three-day recall and 7-days recall. Data was entered in an Excel spread sheet and analyzed using Epi Info version 3.5.1. Proportions were compared using either the chi-square test or Fisher′s exact test. Bivariate analysis was used for identifying variables with potential risk factors on adherence. The differences between the adherent and non-adherent groups were considered to be significant if p &#lt; 0.05.

## Results

A total of 213 caregivers and their children were interviewed. Table 1 shows the general characteristics of the caregivers and the children. There were 109 (51.2%) males and 104 (48.8%) females. Their ages ranged from 5months to 17years with a median age of 6 years. The mean duration of antiretroviral drugs was 38.2±18.9 months (range 3months to 96months). The primary caregiver was the mother in 150 (70.4%). A total of 180 (84.5%) caregivers had at least secondary education and 98 (46%) were older than 35 years. All the caregivers reported having received adherence counseling prior to commencement of the antiretroviral drugs. Ninety-seven (45.5%) children had a co-morbidity which included tuberculosis 64(66%), chronic supurrative otitis media 8(8.2%), dermatitis 7(7.2%), neurological 6(6.2%), renal 6(6.2%), sickle cell anemia 3(3.1%) and asthma 2(2.1%). Four (1.9%) children were on zidovudine/lamivudine/efavirenz combination, 7 (3.3%) were on tenofovir/emtricitabine or lamivudine/ritonavir-boosted lopinavir, 95 (44.6%) were on stavudine/lamivudine/nevirapine combination and 107 (50.2%) were on zidovudine /lamivudine/nevirapine combination. Eighteen (8.4%) children have had their status disclosed to them. Four (22%) were aged between 8 and 9years, 11 (61%) were between 10years and 15years, while 3 (17%) were above 15years. One hundred and ninety-seven (92.5%) caregivers had correct knowledge of the implication of non adherence whereas 16 (7.5%) did not. The commonest identified implications were that child will become ill again (140), virus will be resistant (72), drugs may no longer work (52), child may die from the disease (35) and viral load will rise (18).

**Table 1:** General characteristics of the primary caregivers and the children (n = 213)

Characteristics	Number (%)
**Primary caregiver**	
Mother	150(70.4)
Father	18(8.5)
Others	45(21.1)
**Caregiver's age in years**	
<20	4(1.9)
20-34	111 (52.1)
≥35	98(46)
**Caregiver's education**	
None	3(1.4)
Primary	30(14.1)
Secondary	118(55.4)
Tertiary	62(29.1)
**Gender of child**	
Male	109(51.2)
Female	104(48.8)
**Age of the child**	
<5years	70 (32.9)
5 to <10years	98 (46.0)
> 10 years	45 (21.2)

Table 2 shows that 162 (76.1%) children had adherence rates ≥95%, while 51 (23.9%) had adherence rates less than 95%. Only126 (59.2%) were completely adherent (i.e. took 100% of prescribed drugs in the past one month). The reasons for missing at least one dose of antiretroviral drugs are presented in Table 3. The commonest caregiver-related reasons were forgetfulness 48(55.2%), caregiver travelled 22 (25.3%) and drugs finished 16(18.4%), while the child related reasons were child refused drugs 10(11.5%), child slept 8(9.2%), and child vomited 8(9.2%). On re-assessment of children identified initially as being “non-adherent” (n = 51) after counseling, 49 (96%) of them became adherent (26 = 100%. 18 = 98%, 5 = 95%).

**Table 2:** The adherence score rates of the children and adolescents

Adherence rate (%)	No.	(%)
95 to 100%	162	76.1
90 to <95%	13	6.1
80 to <90%	5	2.3
70 to <80%	13	6.1
<70%	20	9.4
**Total**	**213**	**100**

**Table 3:** Reasons for missing at least one dose of antiretroviral drugs (n = 87)

Reasons	Number	Percent
**Care giver factors**		
Forgetfulness	48	55.2
Care giver travelled	22	25.3
Drugs finished	16	18.4
Caregiver was ill	10	11.5
Too busy work schedule	7	8.0
Took child for spiritual/native healing	7	8.0
Had family problems	5	5.7
Did not understand the dosage	4	4.6
Shared drugs with child	4	4.6
Child was feeling well	3	3.4
No money for transportation	2	2.3
Reduced the dose to last until another refill	2	2.3
**Child factors**		
Child refused drugs	10	11.5
Child slept	8	9.2
Child vomited	8	9.2
Drugs are too many	6	6.9
Child went to school	6	6.9
Drugs are bitter	3	3.4

Sixty eight (31.9%) caregivers reported missing clinic visits. Table 4 shows the reasons for missing scheduled clinic visits. The commonest reasons given were travelled 18(26.5%), caregiver was ill 12(17.6%) and family problems 9(13.2%).

**Table 4:** Reasons for missing clinic visits (n = 68)

Reasons	Number	Percent
Travelled	18	26.5
Caregiver was ill	12	17.6
Family problems	9	13.2
No money for transportation	6	8.8
Child had exams	6	8.8
Still had drugs	6	8.8
Doctor's strike	6	8.8
Unable to get permission from work place	4	5.9
Shared drugs with child	4	5.9
Child was not feeling sick	2	2.9
Taking native medication	2	2.9
Unable to get permission from school authorities	1	1.5
Believe in divine healing	1	1.5
Forgot	1	1.5

Table 5 shows the factors influencing adherence. A poor adherence was significantly observed in children when the mother was the primary caregiver (OR 3.32; 95%CI, 1.33-8.67; p = 0.007). Children less than 5years were more likely to be non-adherent than children above 5years of age (OR 2.62; 95%CI, 1.30-5.31; p = 0.006). Presence of a co-morbidity during follow up was significantly associated with a poorer adherence (OR 3.97; 95%CI, 1.92-8.33; p = 0.0001). On the other hand, caregivers who had a method of remembering medication dosing had a significantly better adherence than those who had no medication reminder strategies (OR 6.34; 95%CI, 3.04-13.31; p = 0.000). Adherence was also better in children with regular scheduled follow up clinic visits (OR 8.55; 95%CI 4.01-18.45; p = 0.000) and those who have had their status disclosed to them (p = 0.008). The caregivers age (p= 0.11), level of education (p = 0.86) and socioeconomic status (p = 0.89) did not predict adherence. The gender of the child (p = 0.84), type of ART drug (p = 0.2) and duration of ART (1.0) also did not significantly affect adherence.

**Table 5:** Factors influencing adherence to antiretroviral treatment in 213 children and adolescents

Factors	Number (n = 213)	Adherent (n = 162)	Non-adherent (n = 51)	OR(95%CI)^a^	P
**Primary caregiver**					
Mother	150(70.4%)	106(70.7%)	44(29.3%)		
Others^b^	63(29.6%)	56(88.9%)	7(11.1%)	3.32(1.33-8.67)	0.007^c^
**Caregiver's age**					
<35 years	115(54%)	82(71.3%)	33(28.7%)		
≥35 years	98(46%)	80(81.6%)	18(18.4%)	1.79(0.89-3.62)	0.11
**Caregiver's education**					
None/Primary	33(15.5%)	26(78.8%)	7(21.2%)		
Secondary/Tertiary	180(84.5%)	136(75.6%)	44(24.4%)	0.83(0.30-2.19)	0.86
**Socioeconomic status**					
High/Middle	125(58.7%)	95(76%)	30(22%)		
Low	88(41.3%)	67(76.1%)	21(23.9%)	1.01(0.51-2.01)	0.89
**Have a method of remembering medication intake**					
No	66 (31%)	34(52%)	32 (48%)		
Yes	147(69%)	128 (87%)	19 (13%)	6.34(3.04-13.31)	0.000^c^
**Gender of child**					
Male	109 (51.2%)	84(77.1%)	25(22.9%)		
Female	104 (48.8%)	78(75%)	26(25%)	0.89(0.45-1.76)	0.84
**Age of the child**					
<5years	65(30.5%)	41(63.1%)	24(36.9%)		
>5years	148(69.5%)	121(81.8%)	27(18.2%)	2.62(1.30-5.31)	0.006^c^
**Duration of antiretroviral drugs**					
<1 year	23(10.8%)	18(78.3%)	5(21.7%)		
>1year	190(89.2%)	144(75.8%)	46(24.2%)	0.87(0.27-2.67)	1.0
**Type of antiretroviral drug**					
First line	206(96.7%)	155(75.2%)	51(24.8%)		
Second line (PI-based)^d^	7(3.3%)	7(100%)	0(0%)	undefined	0.201^e^
**Disclosure**					
No	195 (91.5%)	144 (73.8%)	51(26.2%)		
Yes	18 (8.5%)	18(100%)	0(0%)	undefined	0.008^c^^e^
**Presence of co-morbidity**					
Yes	97 (45.5%)	61(63%)	36(37%)		
No	116 (54.5%)	101 (87%)	15 (13%)	3.97(1.92-8.33)	0.0001^c^
**Missed clinic attendance**					
Yes	68 (32%)	33(48.5%)	35(51.5%)		
No	145 (68%)	129(89%)	16(11%)	8.55(4.0118.45)	0.0000^c^

a Odds ratio (95% confidence interval)

b Includes father, grandmother, uncle, aunt, nanny and elder sibling

c p-value <0.05

d PI = protease inhibitor

e p-value for Fisher's exact test used where a cell is <5

## Discussion

Antiretroviral therapy (ART) success hinges on adherence. Adherence is the extent to which a client's behavior matches the prescribed health care regimen in terms of care (correct date and time for clinic appointment) and treatment (correct drug, timing, dosing, compliance to food restrictions and no missed doses). This is determined through a shared decision making process between the client and health care provider. Unlike drugs for other chronic illnesses where adherence levels of 70-80% are considered adequate to achieve treatment goals, in the case of antiretroviral therapy, adherence levels greater than 95% is required to obtain a successful treatment outcome [2, 8]. An adherence rate below 95% is associated with increasing levels of virologic failure [2]. There is no “gold standard” for assessing adherence and rates may differ depending on the method used. Generally, microelectronic monitoring system devices, plasma drug level measurement and unannounced pill counts although more objective are either very expensive, require laboratory testing and trained personnel or are too intrusive and cumbersome and thus impractical in clinic settings. Patient self-report on the other is a relatively simple, reliable and efficient method of assessing adherence in clinical practice and was the most commonly used method of adherence measurement in many studies [5, 10, 11, 13–15]. It is worth noting that self reporting is subject to bias and that combination of methods (like remaining pills count in addition) would provide more reliable results.

The average rate of adherence varies by the method used to assess it and the group studied, but appears to be approximately 70% [16]. In this study, 76.1% achieved ≥95% adherence which was better than the 49.2% initially reported from the same centre as the present study but in adult population [10]. The adherence rate in this study was comparable to the rate of 75.3% in South East Nigeria [11] and 75.7% in Ethiopia [13] when the combined indicators of correct dose, timing and compliance to food restrictions were considered.

It was however lower than that reported for HIV-infected adults in rural China [17] and HIV infected children in North West Nigeria [14] and Jamaica [18]. The studies in children used small sample sizes (less than 65) and adherence was assessed over three or four days [17, 18] with almost half of the children receiving directly observed therapy [18]. Adherence levels are usually lower when patients are required to recall taking their medications over longer intervals. The adherence rate in this study was higher than rates reported in other studies in Africa [19, 20] and developed countries [3, 5, 6, 16, 21]. The different adherence rates may be as a result of the population studied (adult vs children), adherence assessment methodology (self-report or biological markers) and the definition of adherence rates.

The identified reasons for non-adherence were mainly caregiver factors and included forgetfulness, travelling and drugs finishing and to a less extent, child related factors like refusing drugs, slept and vomited. This also agreed with the report in Jamaica [18] that factors associated with non-adherence in children were primarily caregiver related, however long work hours was the common caregiver factor they identified. This was in contrast to the report by Mukhtar et al [14] who identified financial constraints, unavailability and inaccessibility to medications to be the most common reasons for non-adherence with very few reporting forgetfulness and travelling as the reason for non-adherence. This may be due to the fact that their patients had to purchase the ARV drugs themselves whereas in our centre, the ARV drugs are subsidized and given free to the patients. In a review of 13 studies on adherence to ART in Nigeria, the most prevalent barriers to patient adherence were the cost of therapy, medication side effects, unavailability of ARV drugs and the stigma of taking the drugs [9]. Most of those studies were carried out in adults. As with this study, forgetfulness [11, 13, 17, 19] and travelling [13, 19, 20] were also the most frequently reported reasons for missing doses in some adult studies.

In this survey, children with mothers as the primary caregiver were three times more likely to be non-adherent. This may be due to the mother being too ill with her own illness as majority of paediatric HIV is acquired vertically by mother-to-child transmission [22]. Also, some mothers may not have disclosed their status and that of the child to other family members hence none to assume responsibility for administration of drug to the child when the mother is ill. It may also be that when the mother had died from HIV-related illness, there is usually a concerted effort by other family members to save the child by giving the drugs more regularly.

Children less than five years of age were over two times more likely to be non-adherent. This may be because of their complete dependence on an adult caregiver who may be preoccupied with other duties, and the difficulty in administering drugs to this age group. Older children may be more motivated to assume responsibility for their medication intake. This was at variance with the finding by White et al [18] which showed non-adherence to be significantly correlated with increasing age of the child.

Although majority of the caregivers had at least secondary education and had knowledge of the implications of poor adherence, this did not translate to improved adherence rates. Other studies [23] also showed that level of education did not affect adherence level although lower literacy may impede adherence by interfering with the caregiver's ability to understand the importance of adherence or the specifics of medication dosing. In this study, 7.5% of caregivers did not know the implication of non-adherence while almost 5% of the caregivers who missed doses did not understand the dosage. The socioeconomic status of the caregivers was also not significantly associated with poor adherence. This was the finding in other studies [14, 23, 24].

Those that had a method of remembering drug doses were six times more likely to be adherent. This has also been collaborated by other studies [13, 17] which found having a social support and the use of memory aids to be independent predictors of adherence. Alarms and treatment buddies have been encouraged in some settings as a way of improving adherence [25].

Regular clinic visits encourages positive living, ensures regular drug refills and thus improves adherence to ART. This was confirmed in this study as those children who were adherent to scheduled clinic visits were eight times more likely to be adherent. Whyte et al [18] also reported that non-adherence was positively correlated with missing clinic appointments.

Presence of co-morbidity was associated with poorer adherence. This was not surprising because co-morbidity means increased pill burden, drug-drug interactions and more adverse drug effects all of which are capable of hindering adherence to antiretroviral drugs. The availability of paediatric fixed dose combinations of antiretroviral agents has reduced pill burden and simplified dosing schedules.

Children who have had their status disclosed to them had a better adherence. This may be because resolution of disclosure issues in the household improved family support and adherence strategies devised by families.

The survey had some limitations. The use of self report may overestimate adherence as patients may not accurately remember medication intake over a long time or may exaggerate it in order to impress the health care provider and avoid criticism. Secondly, we have only assessed the level of adherence in terms of missing doses and did not take into consideration correct intake of medication in terms of correct time and observance of dietary restrictions which will all affect drug levels. Thirdly, it was not possible to corroborate adherence with adequate viral suppression. HIV viral load testing is not available in our centre and although absolute CD4+ T-cell lymphocyte counts were used to monitor response to treatment, it is age dependent with younger children having higher counts such that an absolute CD4+ T-cell lymphocyte counts that will be considered normal in older children will suggest severe immunosuppression in children less than 5 years. Again, since the CD4+ T-cell lymphocyte counts gradually fall to adult levels by 5 years, a falling CD4+ count in children less than 5 years does not necessarily mean worsening condition. Immune categorization is better made with CD4 percentage in children less than 5 years.

## Conclusion

Although all the caregivers reported receiving adherence counseling prior to commencement of therapy, adherence is still far from optimal. Barriers to optimal adherence in children are largely caregiver-dependent. Clinicians can support and improve medication adherence by identifying and addressing these barriers to adherence in each particular caregiver-child pair prior to starting therapy and as a continuous process throughout treatment. Self-report of adherence should be assessed regularly at each clinic visit in an open, nonjudgmental manner as a way of enhancing confidence and trust and improving patient outcome. It is worth mentioning that combination in methods for adherence assessment will yield more reliable results.

## References

[1] Panel on Antiretroviral Therapy and Medical Management of HIV-Infected Children (2012). Guidelines for the Use of Antiretroviral Agents in Pediatric HIV Infection. http://aidsinfo.nih.gov/ContentFiles/PediatricGuidelines.pdf.

[2] Paterson DL, Swindells S, Mohr J (2000). Adherence to protease inhibitor therapy and outcomes in patients with HIV infection. Ann Intern Med..

[3] Watson DC, Farley JJ (1999). Efficacy of and adherence to highly active antiretroviral therapy in children infected with human immunodeficiency virus type 1. Pediatr Infect Dis J..

[4] Steele RG, Grauer D (2003). Adherence to antiretroviral therapy for pediatric HIV infection: review of the literature and recommendations for research. Clin Child Fam Psychol Rev..

[5] Gifford AL, Bormann JE, Shively MJ, Wright BC, Richman DD, Bozzette SA (2000). Predictors of self-reported adherence and plasma HIV concentrations in patients on multidrug antiretroviral regimens. J Acquir Immune Defic Syndr..

[6] Murphy DA, Wilson CM, Durako SJ, Muenz LR, Belzer M (2001). Antiretroviral medication adherence among the REACH HIV-infected adolescent cohort in the USA. AIDS Care..

[7] Reddington C, Cohen J, Baldillo A (2000). Adherence to medication regimens among children with human immunodeficiency virus infection. Pediatr Infect Dis J..

[8] Bangsberg DR, Perry S, Charlebois ED (2001). Non-adherence to highly active antiretroviral therapy predicts progression to AIDS. AIDS..

[9] Monjok E, Smesny A, Okokon IB, Mgbere O, Essien EJ (2010). Adherence to antiretroviral therapy in Nigeria: an overview of research studies and implications for policy and practice. HIV/AIDS - Research and Palliative Care..

[10] Nwauche CA, Erhabor O, Ejele OA, Akani CI (2006). Adherence to antiretroviral therapy among HIV-infected subjects in a resource limited setting in the Niger Delta of Nigeria. Afr J Health Sci..

[11] Uzochukwu BSC, Onwujekwe OE, Onoka AC, Okoli C, Uguru NP, Chukwuogo OI (2009). Determinants of non-adherence to subsidized anti-retroviral treatment in southeast Nigeria. Health Policy Plan..

[12] Oyedeji GA (1985). Socio-economic and cultural background of hospitalized children in Ilesha. Nig J Paed.

[13] Amberbir A, Woldemichael K, Getachew S, Girma B, Deribe K (2008). Predictors of adherence to antiretroviral therapy among HIV-infected persons: a prospective study in Southwest Ethiopia. BMC Public Health.

[14] Mukhtar-Yola M, Adeleke S, Gwarzo D, Ladan ZF (2006). Preliminary investigation of adherence to antiretroviral therapy among children in Aminu Kano Teaching Hospital, Nigeria. Afr J AIDS Res..

[15] Gordillo V, del Amo J, Soriano V, González-Lahoz J (1999). Sociodemographic and psychological variables influencing adherence to antiretroviral therapy. AIDS..

[16] Machtinger EL, Bangsberg DR (2005). Adherence to HIV antiretroviral therapy. HIV In Site Knowledge Base Chapter. http:hivinsite.ucsf.edu/InSite?page=kb-03-02-09.

[17] Wang X, Wu Z (2007). Factors associated with adherence to antiretroviral therapy among HIV/AIDS patients in rural China. AIDS..

[18] White YRG, Pierre RB, Steel-Duncan J (2008). Adherence to antiretroviral drug therapy in children with HIV/AIDS in Jamaica. West Indian Med J..

[19] Potchoo Y, Tchamdja K, Balogou A, Pitche VP, Guissou IP, Kassang EK (2010). Knowledge and adherence to antiretroviral therapy among adult people living with HIV/AIDS treated in the health care centers of the association “Espoir Vie Togo” in Togo, West Africa. BMC Clinical Pharmacology..

[20] Weiser S, Wolfe W, Bangsberg D (2003). Barriers to Antiretroviral Adherence for Patients Living with HIV Infection and AIDS in Botswana. J Acquir Immune Defic Syndr..

[21] Belzer ME, Fuchs DN, Luftman GS, Tucker DJ (1999). Antiretroviral adherence issues among HIV-positive adolescents and young adults. J Adolesc Health..

[22] Eneh AU, Ugwu RO (2007). Paediatric HIV at the University of Port Harcourt Teaching Hospital, Port Harcourt. Nig J Paed..

[23] Müller AD, Bode S, Myer L, Stahl J, von Steinbüchel N (2011). Predictors of adherence to antiretroviral treatment and therapeutic success among children in South Africa. AIDS Care..

[24] Orrell C, Bangsberg DR, Badri M, Wood R (2003). Adherence is not a barrier to successful antiretroviral therapy in South Africa. AIDS..

[25] Wessels X, Nattrass N, Rivett U (2007). Improving the efficiency of monitoring adherence to antiretroviral therapy at primary health care level: a case study of the introduction of electronic technologies in Guguletu, South Africa. Development Southern Africa.

